# Breast Tumor Laterality in the United States Depends Upon the Country of Birth, but Not Race

**DOI:** 10.1371/journal.pone.0103313

**Published:** 2014-08-01

**Authors:** Trevor Sughrue, James P. Brody

**Affiliations:** Department of Biomedical Engineering, University of California Irvine, Irvine, California, United States of America; West Virginia University, United States of America

## Abstract

More breast cancers are diagnosed in the left breast than the right. The ratio (l/r) is called the laterality ratio. We analyzed 1.2 million cases of breast cancer diagnosed in the US between 1973 and 2010 and recorded by the Surveillance, Epidemiology, and End Results (SEER) program. We found that the laterality ratio depends upon the country of birth, but not race of the patient. We identified five countries of birth that had p-values larger than 0.995, while we expected to see less than 1. Those born in Japan (l/r = 1.14, p = 0.997), the Ryukyu Islands (l/r = 2.6, p = 0.998), Laos (l/r = 1.62, p = 0.9999) and Algeria (l/r = 2.1 p = 0.9959) had significantly larger laterality ratios compared to the overall SEER population (l/r = 1.04). Those born in Poland (l/r = 0.92, p = 0.997) had a laterality ratio significantly less than expected. We compared the laterality ratio calculated for tumors occurring in each quadrant of the breast for two immigrant populations: those born in Japan and those born in Poland. We found the only significant difference was in the laterality ratio of the upper outer quadrant. Thus, the birthplace effect appears to only occur in the upper outer quadrant of the breast. Finally, we found a small, but statistically significant, increase in the breast cancer laterality ratio with age, and decrease with birth year and year of diagnosis.

## Introduction

Slightly more breast tumors are diagnosed in the left breast than the right [1]–[5]. Although this fact is well established, it is not widely known.

The breast is a paired organ. The two breasts share many of the risk factors known to contribute to the development of cancer: genetics, environmental exposure, diet, estrogen exposure, etc. By studying differences in the occurrence of breast cancer between the left and right breast, we can control for these common risk factors. Understanding the genesis of the left right asymmetry should lead to a better understanding of how breast cancers develop in general.

Several facts about the asymmetric laterality of breast cancer are known. Previous studies of breast cancer asymmetry have established that the laterality ratio is greater than 1.0 in both women [3] and men [6]. Different quadrants of the breast have different laterality ratios [4]. The laterality ratio may vary with age [6]. Asymmetric laterality is present in both invasive and *in situ* tumors [6].

Possible explanations have included the left breast is slightly larger than the right [7], breast feeding preferentially on the right breast protects from cancer [8], and that right handed women check the left breast for lumps more often [9]. However, these explanations have been countered by findings that different quadrants of the breast have different laterality ratios [4], men also have asymmetric occurrence of breast tumors [6], and this asymmetry is present in both invasive and *in situ* tumors [6].

The most recent comprehensive analysis of the laterality of breast cancer was in 2004 [4]. This 2004 study examined 419,935 cases of breast cancer.

## Results

We examined 1,216,226 cases of breast cancer diagnosed in the United States between 1973 and 2010 and recorded by the Surveillance, Epidemiology, and End Results Program [10]. The most recent previous comprehensive study of breast cancer laterality ratios occurred in 2004 [4]; that study analyzed 419,935 cases of breast cancer. Our study had almost three times as many cases.

**Figure 1:**
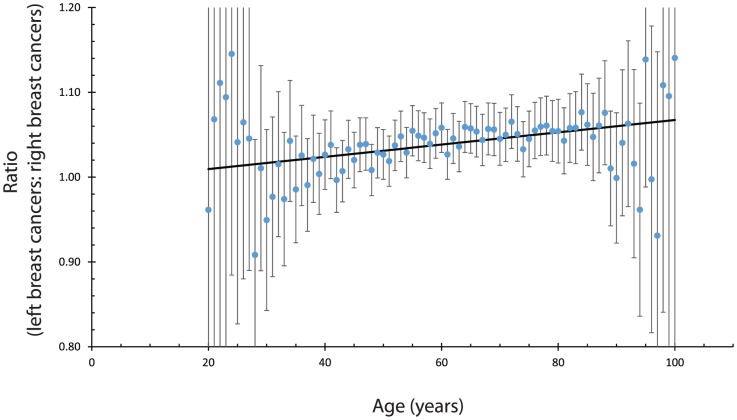
The circles indicate the measured ratio of the number of breast cancers diagnosed in the left breast as compared to the right breast. The error bars indicate 95% confidence intervals. The solid line is the best fit to a linear model. The best fit slope was 0.00087 (95% confidence interval 0.00115, 0.00059) ratio units per year. The probability that this slope would occur by chance, given that the actual slope is 0, is about one chance in 10,000,000.

First, we tested whether the laterality ratio is constant or varies linearly with age, see Figure 1. We found a slight increase in the laterality ratio with age. The best estimate of the slope is 0.00087 ratio units/year of age with a 95% confidence interval of 0.00115 to 0.00059). The linear model had a p-value of 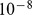 for the probability that the slope is actually zero, or equivalently 0.99999999 for the probability that the slope is not zero.

The data we used was from breast cancers diagnosed between 1973 and 2010, but the number of breast cancers in the data are not equal each year. The SEER network of registries has constantly expanded, more recent years contain more diagnosed cases of breast cancer. Over half of the 1.2 million diagnosed breast cancers in our dataset were diagnosed after 2001.

Although we measured a non-zero variation in the laterality rate with age, this measurement could be an artifact from a period or birth cohort effect. Age, period, and cohorts are related and indistinguishable in a cross-sectional dataset [11], [12].

**Figure 2:**
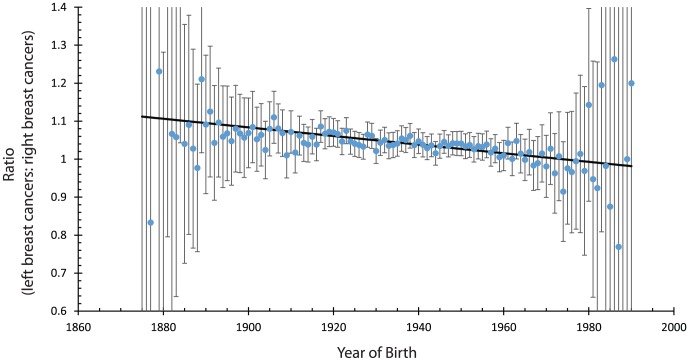
The circles indicate the measured ratio of the number of breast cancers diagnosed in the left breast as compared to the right breast. The error bars indicate 95% confidence intervals. The solid line is the best fit to a linear model. The best fit slope was −0.0012 (95% confidence interval 0.0104, 0.0042) ratio units per year. The probability that this slope would occur by chance, given that the actual slope is 0, is 0.000008, about one chance in 100,000.

We tested whether a birth cohort effect exists by measuring how much the laterality ratio varies with birth year, see Figure 2. The best estimate for the slope was 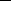
0.00083 ratio units/year with a 95% confidence interval from 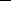
0.00109 to 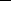
0.00057. This linear model leads to a p-value of 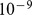 for the probability that the actual slope was zero.

**Figure 3:**
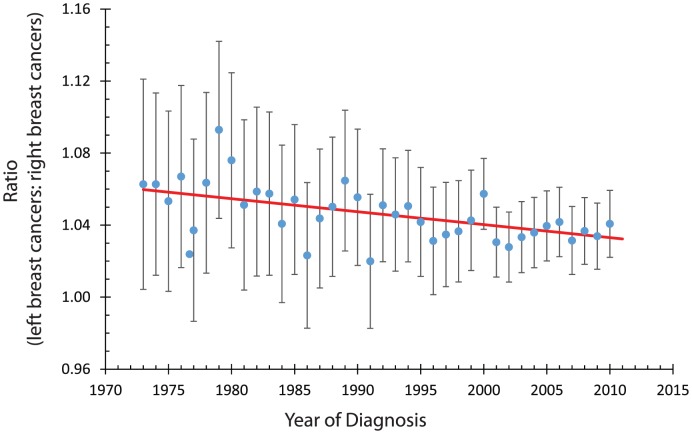
The circles indicate the measured ratio of the number of breast cancers diagnosed in the left breast as compared to the right breast. The error bars indicate 95% confidence intervals. The solid line is the best fit to a linear model. The best fit slope was −0.00072 (95% confidence interval −0.00038, −0.00106) ratio units per year. The probability that this slope would occur by chance, given that the actual slope is 0, is 0.0001, about one chance in 10,000.

We also found a period effect, see Figure 3. We measured a slope of 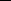
0.00072 ratio units/year (95% CI 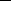
0.00038 to 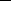
0.00106).

Male breast cancer cases also exhibit asymmetric laterality. We found 4190 tumors diagnosed in the left breast of men, but only 3868 tumors diagnosed in the right breast of men. These data gave a laterality ratio of 1.08 with a 95% confidence interval of (1.03, 1.13), which was significantly different from 1.0. The male laterality ratio is not significantly different than the overall female laterality ratio, which we measured as 1.041 with a 95% confidence interval from 1.036 to 1.046.

Breast cancers do not occur uniformly throughout the breast. The location of a tumor can be described by the quadrant of the breast in which it occurs. Each breast is categorized into one of four quadrants: upper-inner, lower-inner, upper-outer, and lower-outer. In addition, there are three other regions outside these quadrants: the nipple, the areola, and the axillary tail. In the SEER data, about 40% of the tumor locations are recorded as overlapping or not specified. About 33% of the tumors are in the upper outer quadrant, 9% occur in the upper inner quadrant, 5% in the lower inner quadrant and 6% in the lower outer quadrant. The remaining 8% are found in the areola (6%), the nipple (1%) and the axillary tail (1%). These numbers vary slightly between the left and right breast.

**Figure 4:**
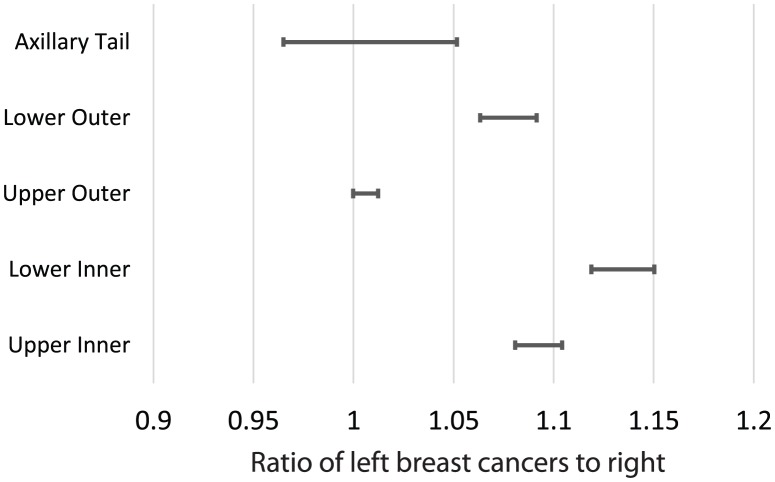
The lines indicate the 95% confidence interval ranges for the laterality, as measured in tumors occurring in different regions of the breast.

We tested whether the laterality ratio was the same in each of the four quadrants of the breast. We found that the laterality ratio varied significantly, depending on the location of the tumor, see Figure 4. The laterality ratio in the upper outer quadrant was slightly above 1.0, while the laterality ratio for the lower inner quadrant was significantly higher, 1.14. The lower outer and upper inner quadrants had laterality ratios in between the two extremes.

We next tested whether the laterality ratio varied with race. The SEER data listed breast tumors occurring in patients classified into 30 different race categories. We would expect about three of these to have p-values greater than 0.90, or six of these categories to exceed the two tailed 90% confidence interval. We found seven, three of which had p-values exceeding 0.95, but none of which exceeded 0.99. Thus, we conclude that the distribution of laterality ratios among race classifications is consistent with random variation and no significant correlation between race classification and laterality ratio is measurable.

The laterality ratio also might vary with birthplace. The SEER database includes information on the place of birth for almost 700,000 patients diagnosed with breast tumors. (About 520,000 of the 1.2 million breast cancer tumors have their birthplace listed as unknown.) Outside of the 50 states, 170 different birthplaces were listed for people who developed breast tumors. We computed the probability (p-value) that the population born in a given place had a significantly different laterality ratio, as compared to the population of the entire SEER database. We found 5 birthplaces that had p-values larger than 0.995, while we expected to see less than 1. Those born in Japan (p = 0.997), the Ryukyu Islands (p = 0.998), Laos (p = 0.9999) and Algeria (p = 0.9959) had significantly larger laterality ratios compared to the overall SEER population. Those born in Poland (p = 0.997) had significantly less than expected laterality ratios. See Table 1.

**Table 1:** Laterality ratio by country of birth.

Country	Right	Left	Ratio	p-value
Laos	62	101	1.6	0.9999
Ryukyu Islands	5	13	2.6	0.9984
Japan	1316	1504	1.14	0.9966
Algeria	9	19	2.11	0.9959
Poland	990	907	0.91	0.9965

**Figure 5:**
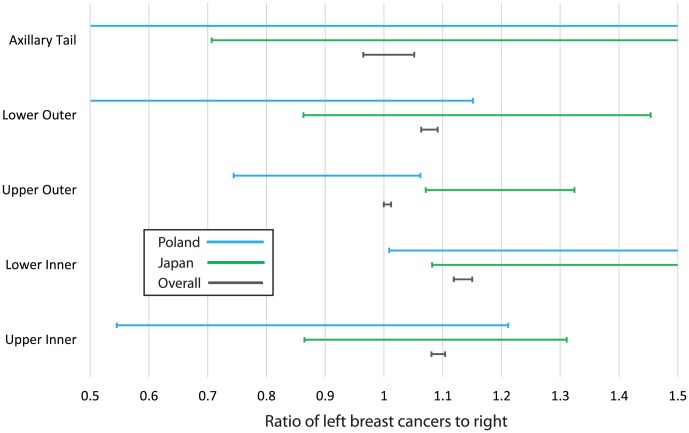
The lines indicate the 95% confidence interval ranges for the laterality, as measured in tumors occurring in different regions of the breast. We compared how these were distributed for people born in Poland, Japan, and the overall population. We found the only significant difference between Poland and Japan occurs in the upper outer quadrant of the breast.

Next we sought to establish whether the difference in birthplace laterality ratios arose from (1) different distributions of where the tumor occurs (primary site or the quadrant of the tumor) or (2) a shift in the laterality ratio of each location where the tumor occurs. First, we compared the distribution of tumor locations (the quadrant of the breast in which the tumors occur) in those born in Poland with those born in Japan. We found that the relative distributions of tumor location were not significantly different. Next, we compared the laterality ratio by tumor location for the two populations. We found the only significant difference was in the laterality ratio of the upper outer quadrant. Thus, the birthplace effect appears to only occur in the upper outer quadrant of the breast. See Figure 5.

## Discussion

Several explanations have been proposed for the excess of left breast tumors to right breast tumors. One suggestion is that the left breast is larger than the right. A study measuring the volume of the two breasts in 598 women found that 54% had a larger left breast, while 46% had a larger right breast [13]. However, this explanation fails to explain why the laterality ratio varies by the quadrant of the breast.

Another proposed explanations is that mothers preferentially use the right breast over the left when breastfeeding and that this protects from breast tumors. However, we have found that male breast cancers also have a laterality ratio significantly greater than one.

A third hypothesis is that right handed women preferentially check the left breast for lumps [9]. If true, then one would expect that the four quadrants of the breast would all have the same laterality ratios and that different laterality ratios would exist between small and large tumors. However, the laterality ratio differs in each quadrants of the breast. Furthermore, small tumors occur with the same laterality ratio as large tumors [4].

We found that the laterality ratio increases with age, Figure 1. This factor has been investigated previously. One study published in 1996 [3] noted a non-zero slope for the laterality ratio vs age function, but did not specify the specific value they measured. However, a 2004 study [4] saw no effect of age on the laterality ratio, but also did not give a slope and confidence interval. This [4] 2004 study only looked at a small time period (4 years), which would reduce the effect of period variations.

Some [6] have suggested the age effect is better described with two age categories, pre menopause and post menopause. According to this suggestion, the pre menopause category would have symmetric laterality (l/r = 1) while the post menopause age group would have asymmetric laterality (l/r¿1). We found no evidence for this hypothesis.

The laterality ratio decreases slightly with birth year. This effect has not been reported in previous studies of laterality in breast cancers. We also noted a slight decrease in the laterality ratio with the year of diagnosis.

Although we noted statistically significant changes in the laterality ratio with age, year of birth, and year of diagnosis, we cannot conclude that any of these three factors have an effect on the laterality ratio. Age, year of birth (cohort), and year of diagnosis (period) are linearly related. Thus, the linear regression coefficients are not unique [14]. This result is part of the well studied age-period-cohort identity problem [15].

The laterality ratio varies by the quadrant in which the tumor is found, Figure 4. This finding supports results previously reported [4]. [4] noted that the laterality ratio for the upper outer quadrant was consistent with 1.0, while the other three quadrants were consistent a single value of about 1.09. We measured the laterality ratio of the upper outer quadrant (1.006, 95% confidence interval 1.000–1.012) as larger than 1.0 (p = 0.95) and the lower inner quadrant (1.13) higher than the other two quadrants. Our 95% confidence intervals are smaller than, but overlap with those of [4].

The laterality ratio varies with country of birth, but not race. This effect has not been reported before. The people diagnosed in this study were all living in the United States at the time their breast tumor was found. A small percentage were born in other countries, but no information is available on when they immigrated to the US. This effect implies that some factors early in life contribute to the laterality ratio. This variation in the laterality ratio occurs through changes in the laterality of tumors found in the upper outer quadrant.

Environmental stimuli in the uterus during development may cause permanent changes that lead to chronic disease later in life, according to the developmental origin of disease hypothesis [16], [17]. Developmental factors could have a major role in breast cancer [18]–[20]. For instance, (1) significant variation of national breast cancer rates [21], (2) age-specific incidence rates that decrease with age indicate that breast cancer only occurs in a limited population [22], [23] and (3) birth weight [24]–[26] is correlated with breast cancer risk.

The left/right asymmetry is dependent on the location in the breast in which it occurs. The left:right ratio is dependent on location of birth. Those born in Japan have a significantly higher ratio, while those born in Poland have a significantly lower ratio. The left-right ratio shows a slight dependence on age at diagnosis, year of birth, and year of diagnosis. However, these three parameters are related and it is not possible to definitively determine if any one of these factors effect the left-right ratio. These findings are consistent with a causative process in development for breast cancer, but are inconsistent with the hypothesis that the excess number of tumors found in the left breast is because the left breast is larger.

In conclusion, any proposed hypothesis to explain the left-right asymmetry in breast cancer would need to answer the following questions: Why does the laterality vary by quadrant of the breast, from close to 1.0 in the upper outer quadrant to 1.14 in the lower inner quadrant? Why does the laterality ratio depend upon the country of birth for people living in the United States? Why does the asymmetric laterality of breast cancer also occur in men?

## Materials and Methods

We used the Surveillance, Epidemiology and End Results (SEER) Program data, November 2012 submission. This data includes cases reported from 1972 through 2010. We downloaded the case files and loaded them into a SQLite database using a custom Python program. We queried this database using SQL commands to obtain the statistics presented here.

The SEER Program is a consortium of (currently) 19 different cancer registries. These registries code all tumors diagnosed within specific geographic areas according to standardized SEER guidelines. The coding guidelines for breast cancer describe how to consistently code the primary site, grade, and laterality of a breast tumor. The SEER program checks the submissions from the different registries to ensure quality and consistency and releases data annually.

The data was anonymized and de-identified by the SEER Program before we had access to it. All personal identifiable information was removed including names, birthdates, social security numbers, and contact information. We signed the Surveillance, Epidemiology, and End Results Data-Use Agreement to obtain access to the data. This agreement further ensures patient privacy by restricting the distribution of the raw data to others who also sign the Data-Use Agreement. Individual patient consent was neither required nor available.

We measured the laterality ratios as the number of primary breast tumors diagnosed in the left breast divided by the number of primary breast tumors diagnosed in the right breast. We identified about 1.2 million primary tumors classified as breast (013) by their Collaborative Stage Schema values. Of these 1.2 million, about 13,000 (or about 1%), were either missing information on which breast it occurred, or classified as bilateral from a single primary tumor. These tumors not classified as being in either the left or the right breast were excluded from this analysis.

For each person diagnosed with a breast tumor, we recorded the age at diagnosis, sex, year and place of birth. We also recorded the location of the tumor within the breast (classified by quadrant) and the year in which the tumor was diagnosed.

We computed 95% confidence intervals assuming the number of breast cancers followed the Poisson distribution. Specifically, we used a normal distribution approximation; we calculated the 95% confidence interval as 1.92 times the standard deviation. The standard deviation was computed as the square root of the raw number of counts. Confidence intervals in ratios and sums were calculated using standard propagation of errors.

The significance of differences between groups were quantified using p-values. These p-values were computed assuming a normal distribution of counts of breast cancer cases.

Linear fits to the data were computed with the statistical computing environment R using the function lm(). This function was called with the weights being equal to the inverse of the variance, which is the number of breast tumors diagnosed, for each category.
